# Implicit theories of leadership malleability: How beliefs about the malleability of leadership characteristics predict employee voice

**DOI:** 10.1371/journal.pone.0351187

**Published:** 2026-06-11

**Authors:** Ziyi Zhang, Michael P. Haselhuhn

**Affiliations:** 1 The Hong Kong Polytechnic University, Hung Hom, Kowloon, Hong Kong; 2 University of California, Riverside, Riverside, California, United States of America; The Open University of Israel, ISRAEL

## Abstract

Despite the proliferation of research emphasizing the importance of growth mindsets in a wide range of domains, research examining these mindsets in the context of leadership has been overlooked. This research expands implicit theories to the leadership discipline and explores how beliefs about the fixedness or malleability of leadership characteristics (termed *Implicit Theories of Leadership Malleability; ITLMs*) affect employee voice. Through four studies, findings suggest that beliefs in the malleability of leadership characteristics predict employees’ voice behaviors in the workplace, and that this link is mediated by the perceived efficacy of voice. The relationship between ITLMs and voice is stronger when voice is directed toward growth and improvement (i.e., promotive voice) relative to voice directed toward avoiding failure (i.e., prohibitive voice).

## Introduction

Organizations invest more than $60 billion annually in leadership development initiatives [1], yet many organizations simultaneously invest heavily in selecting individuals with innate leadership potential [2–6]. This paradox reflects a fundamental question that has long occupied leadership scholars: Are effective leaders born or made? Both perspectives appear to have some merit (e.g., [7,8]), suggesting that there may be a great deal of variance in individuals’ views of leadership malleability. Indeed, understanding these differing beliefs is important, as they can shape how people approach leadership development, respond to challenges, and interact within organizational contexts.

This research examines how people’s implicit beliefs regarding the fixedness or malleability of leadership characteristics affect important organizational outcomes, specifically employee voice behavior, shedding light on the practical implications these beliefs hold for management practice and organizational effectiveness. Drawing on Dweck’s influential work on implicit theories (i.e., growth mindsets, [9,10]), this framework is applied to the domain of leadership, which are referred to as Implicit Theories of Leadership Malleability (ITLMs). Following Dweck’s framework, a distinction is drawn between two types of ITLMs: *entity* beliefs, which assume that leadership characteristics are stable, innate, and fixed; and *incremental* beliefs, which assume that leadership characteristics are malleable, learnable, and capable of developing over time. In this research, leadership characteristics are conceptualized as the underlying attributes and tendencies that shape how leaders behave and interact with followers, which in turn give rise to observable leadership styles and downstream organizational outcomes [11–13]. These characteristics are reflected in the specific actions leaders take in managing others. For example, some leaders delegate decision-making authority to subordinates and encourage participation, whereas others retain control and centralize decisions [14,15]. Such differences in enacted behaviors illustrate how abstract leadership characteristics manifest in concrete leadership practices, thereby making them a meaningful target of individuals’ implicit beliefs.

Prior research on implicit theories, rooted in Dweck’s foundational framework [6, 9], distinguishes between incremental and entity beliefs regarding the malleability of personal attributes. Individuals endorsing incremental theories view attributes such as intelligence as developable, whereas those endorsing entity theories regard them as fixed [16,17]. These beliefs systematically shape goal orientation and motivation, with incremental theorists more likely to pursue learning goals oriented toward mastery and development, and entity theorists more likely to adopt performance goals focused on demonstrating competence and avoiding negative evaluations [10,18–20]. As a result, learning-oriented individuals tend to exhibit persistence, adaptive coping, and challenge-seeking, whereas performance-oriented individuals are more prone to avoidance, negative self-evaluations, and disengagement following setbacks [9,21–23]. Although initially developed in educational contexts, these patterns have been extended to organizational settings, where growth mindsets are associated with adaptability, learning, and sustained performance, while fixed mindsets are linked to defensiveness, feedback avoidance, and maladaptive responses under evaluative pressure [24–27].

Despite these advances, the literature has paid limited attention to how implicit theories operate when the focal attribute shifts from general personal qualities to leadership characteristics. Existing research has predominantly examined domain-general implicit beliefs or focused on leaders’ own mindsets about others (e.g., employee potential), leaving the role of implicit beliefs about leadership itself underexplored. This gap is theoretically meaningful because leadership is inherently relational and socially constructed, suggesting that beliefs about the malleability of leadership may shape how individuals interpret leader behavior and regulate their own responses in ways that differ from self-focused domains. Moreover, prior work has not sufficiently considered how such beliefs function from the followers’ perspective, particularly in shaping proactive and discretionary behaviors in organizations. By extending implicit theories to the leadership domain and conceptualizing Implicit Theories of Leadership Malleability (ITLMs), the present research addresses this gap and develops a more context-specific account of how malleability beliefs influence employee behavior.

Understanding ITLMs is important because both leaders and followers hold these beliefs, and each set of beliefs could independently or interactively affect organizational behavior in myriad ways. ITLMs can be studied from the leaders’ perspective, influencing how leaders develop leadership skills; from the followers’ perspective, shaping how followers interpret and respond to their leaders’ behaviors; and through the interplay between leaders’ and followers’ ITLMs. This research begins by focusing specifically on the followers’ perspective because followers’ perceptions and interpretations fundamentally determine how effectively they respond to leadership initiatives and engage in leader-follower interactions. Thus, understanding followers’ ITLMs is crucial for revealing insights into their willingness to invest in leader-follower relationship, and ultimately, their contribution to organizational effectiveness.

Specifically, the current research examines followers’ willingness to engage in *voice*, defined as discretionary behavior that employees use to express constructive opinions, concerns, or ideas about work-related issues [28,29]. Voice is an important behavior to understand, because it can spark organizational innovation, facilitate organizational adaptation to competition, and positively influence organizational effectiveness (e.g., [30–32]). However, not all followers may perceive engaging in voice as equally worthwhile. Drawing on the implicit theories framework, it is proposed that followers with incremental ITLMs, who view leadership characteristics as malleable, will be more likely to view their leaders as receptive to suggestions and capable of growth or change. Consequently, these followers will perceive constructive interactions with their leaders as more efficacious and valuable. This perception, in turn, increases followers’ willingness to engage proactively in voice behaviors. Conversely, followers who hold entity ITLMs are likely to perceive leadership traits as fixed and thus see less benefit in providing input, ultimately reducing their willingness to engage in voice.

This work makes several contributions to literature on leadership, voice and growth mindsets in general. First, applying the implicit theories framework specifically to the domain of leadership adds to our knowledge of the psychology of leadership and followership [33,34]. Second, this research joins a small body of work that examines the social-cognitive antecedents of employee voice (e.g., [35]). Although most research on voice focuses on how organizational conditions and leaders’ traits can facilitate employee voice (e.g., [36,37]), this work illustrates the importance of understanding followers’ beliefs and perceptions as well. Finally, this research extends existing research on growth mindsets to the leadership domain. By doing so, new avenues are opened for understanding how implicit theories of leadership characteristics can direct employees’ behaviors within organizations.

### Implicit Theories of Leadership Malleability (ITLMs)

Dweck and her colleagues identified different implicit theories that people hold about the malleability of personal attributes [9]. Individuals who hold incremental theories (i.e., a growth mindset) view personal attributes as malleable, controllable, and subject to change through effort and experience. Conversely, those holding entity theories (i.e., a fixed mindset) view personal attributes as fixed, stable and impossible to control [16,17]. These implicit theories have been studied across numerous domains, including beliefs about intelligence, personality, and moral character, consistently demonstrating their significant implications for personal motivation, learning processes, and behaviors (e.g., [38–42]).

More recently, researchers have extended implicit theory research to the organizational context and have found that individuals’ implicit theories are influential in understanding organizational behavior. For example, Kray and Haselhuhn [43] demonstrated that negotiators holding incremental beliefs about negotiation ability perform better, as these individuals persist and adapt more effectively when faced with challenges. Similarly, research by Heslin and colleagues [24,44] found that leaders with incremental theories about personality were more attentive to changes in followers’ performance, demonstrating greater responsiveness and adaptability in their leadership approaches. From an employee perspective, a recent meta-analysis suggested that organizational citizenship behavior and task performance are both positively related to employees’ growth mindsets [45].

Additionally, research has explored implicit theories at the organizational level, studying how organizational mindsets working as a collective mindset (i.e., organizational cultures endorsing incremental or entity theories about a particular domain; [46,47]) affect individuals’ behavior. Murphy and Dweck [47], for instance, showed that employees who seek organizational acceptance pay attention to organizational mindsets, adjusting their behaviors and self-presentation accordingly. Although this research has established the relevance of implicit theories in an organizational context and highlighted the importance of implicit theories within organizational contexts [26,27], there remain critical areas, such as leadership characteristics, that have yet to be fully explored.

Importantly, the current focus on implicit theories of leadership differs conceptually from the dominant approaches in the leadership literature that emphasize specific leadership styles or behaviors, such as transformational or transactional leadership. However, rather than positioning these perspectives as competing explanations, we conceptualize implicit theories of leadership malleability (ITLMs) as a higher-order cognitive framework that shapes how individuals interpret and respond to these established leadership behaviors. Rather than examining what leaders do, ITLMs capture individuals’ meta-beliefs about whether leadership characteristics themselves are fixed or malleable. For example, ITLMs influence the extent to which followers view transformational leadership behaviors (e.g., vision articulation, individualized consideration) as signals of stable leader traits versus developable competencies, thereby shaping the effectiveness of such behaviors. In this sense, ITLMs operate at a more fundamental, cognitive level, shaping how individuals interpret, evaluate, and respond to leadership phenomena. This perspective is consistent with social cognition approaches to leadership, which emphasize that leadership is not solely a function of objective behaviors but is co-constructed through followers’ perceptions and interpretive frameworks [33,48–50]. Extending this logic, for example, ITLMs also provide a cognitive foundation for relational frameworks such as leader–member exchange (LMX). Specifically, followers who endorse a malleable view of leadership may be more likely to interpret leader behaviors as opportunities for relationship development, thereby facilitating higher-quality exchanges, whereas entity-oriented followers may perceive such interactions as diagnostic of fixed relational standing. By focusing on beliefs about the developability of leadership, ITLMs complement existing leadership theories by identifying a higher-order lens through which leadership behaviors are construed.

Aligning with the extensive evidence from other domains and recognizing the significant yet understudied potential in leadership contexts, the current research applies the implicit theories framework explicitly to leadership characteristics. Building on the implicit theories of personality construct [10,17], this research considers a continuum of beliefs, anchored at one end by individuals who predominantly endorse the theory that leadership characteristics are inherently stable and fixed (i.e., entity theorists), and at the other end by individuals who predominantly endorse the theory that leadership characteristics are malleable and responsive to growth through effort, training, or experience (i.e., incremental theorists).

Applying implicit theories to leadership domain provides several key insights relevant to organizational functioning. Importantly, leader-follower interactions do not solely depend on leaders’ traits or behaviors; they are also significantly shaped by followers’ subjective perceptions and interpretations of leadership itself (e.g., [48,51]). Thus, exploring followers’ ITLMs is particularly crucial, as followers’ beliefs can significantly influence their motivation, engagement, and behavioral responses to leaders. Followers who hold incremental beliefs about leadership may perceive their leaders as more receptive and open to constructive dialogue, motivating them to actively participate in organizational improvement efforts. Conversely, followers with entity beliefs might hesitate to engage proactively, perceiving limited efficacy or potential risks in voicing suggestions or concerns.

The followers’ perspective is prioritized here because followers’ implicit theories fundamentally shape the nature and quality of their interactions with leaders. The follower perspective provides valuable insights into leader-follower dynamics, as followers’ interpretations and responses to leadership attempts critically determine the effectiveness of leadership and organizational outcomes [33,34,48,49,52,53]. Specifically, this research examines how followers’ ITLMs influence their willingness to engage in voice behaviors—expressing constructive concerns, suggestions, or innovative ideas to leaders.

### ITLMs and employee voice

Workplace voice has long been considered to be beneficial to organizations, and employee voice has been identified as an effective strategy used by employees to deal with workplace issues and correct organizational errors [31,54–58]. Employee voice contributes to management decision-making and facilitates organizational adaptation to competition, which is beneficial to the organization’s working efficiency [59–64]. Studies also show the benefits of voice for individual employees, as employees feel more valued and have a stronger sense of control by engaging in voice behaviors [65,66].

Despite the benefits of voice, it is an underused strategy by employees when faced with organizational issues. Welch and Welch [67] suggested that voice is in fact stifled in many organizations and employees are often hesitant to engage in voice, particularly when the information could be viewed by the recipient as negative [68]. In the leader-follower relationship, followers usually think about whether the leader would take their suggestions seriously before they voice their opinions because even leaders who are extremely open to employees’ ideas may still feel vulnerable [69]. Thus, employees who engage in voice face risks, such as low performance ratings, which makes them consider whether expressing voice is worthwhile before they engage in voice behaviors [62,70]. Therefore, employees are more likely to engage in voice when voice is perceived as more efficacious [37,62]. In other words, one of the most important reasons that employees “keep their mouths shut” is that they may be uncertain about whether managers will actually endorse their suggestions [61,62]; conversely, employees are more likely to speak up if they expect their voice could actually make a difference [70–73].

To more precisely explain why ITLMs should influence perceived efficacy of voice, the current research draws on a social-cognitive perspective of expectancy formation. In leader–follower interactions, employees do not decide whether to speak up based solely on objective conditions; rather, they form expectations about whether their input will be taken seriously and lead to meaningful change [72,73]. These expectations are shaped by underlying beliefs about whether leaders are capable of change. Thus, beliefs about the malleability of leadership characteristics provide a critical cognitive basis for evaluating the likely effectiveness of voice.

The established links between the perceived efficacy of voice and voice behaviors suggest that factors affecting the perceived efficacy of voice may also ultimately affect individuals’ willingness to engage in voice. Here, it is proposed that ITLMs shape perceived efficacy of voice by influencing followers’ expectations about leaders’ responsiveness to input. Incremental theorists, who view leadership characteristics as malleable, are more likely to believe that leaders can learn, adapt, and improve in response to feedback. As a result, they are more likely to expect that expressing suggestions or concerns will lead to meaningful changes in leadership behavior. This expectation directly enhances the perceived efficacy of voice, as voice is seen as a viable mechanism for influencing leadership.

In contrast, entity theorists, who view leadership characteristics as fixed, are less likely to believe that leaders will change in response to feedback. From this perspective, even well-intentioned voice is unlikely to produce meaningful outcomes, as the target of influence is perceived as relatively immutable. Consequently, entity theorists are more likely to discount the effectiveness of voice, perceiving it as unlikely to make a difference.

In sum, the current research argues that ITLMs shape voice behavior by influencing followers’ beliefs about whether voice can effectively make a difference. Individuals who endorse incremental ITLMs are more likely to expect that leaders can improve, thereby increasing the perceived efficacy of voice and encouraging speaking up. In contrast, those individuals with fixed ITLMs do not believe their leaders can improve or change, reducing the perceived utility of voice and discouraging engagement. Thus, perceived efficacy of voice serves as a key psychological mechanism linking ITLMs to employee voice behavior. Formally, two predictions are made:

*Hypothesis 1:* Followers’ ITLMs associate with their tendency to engage in voice behaviors, such that stronger incremental ITLMs will be associated with a greater tendency to engage in employee voice.

*Hypothesis 2:* Perceived efficacy of voice will mediate the relationship between ITLMs and employee voice.

### Promotive voice and prohibitive voice

While the reasoning above provides an important foundation for understanding the impact of ITLMs, employee voice itself can manifest in different forms, each with distinct implications and psychological antecedents. Thus, to achieve a more comprehensive understanding, the current work considers how the specific type of voice behavior may moderate the relationship between followers’ ITLMs and their voice behaviors. Specifically, recent research has identified two dimensions of employee voice: Promotive voice, or employees expressing ideas and suggestions to improve organizational functioning, and Prohibitive voice, or employees expressing concerns and worries to prevent organizational failure [60,74]. The distinction between promotive voice and prohibitive voice has gained empirical support in multiple studies examining voice in organizational contexts [35,74–76], highlighting the usefulness of differentiating these dimensions to better understand antecedents and consequences of employee voice.

Although both promotive and prohibitive voice involve challenging the organizational status quo and are oriented toward benefiting the organization [77], they differ notably in their behavioral content, underlying motivations, and psychological implications [60]. Promotive voice focuses on positive suggestions and ideas intended to enhance or optimize current processes, often signaling an employee’s belief in continuous organizational improvement [77,78]. Prohibitive voice, on the other hand, typically addresses existing or anticipated problems, highlighting potential threats or errors that could undermine organizational effectiveness. Consequently, the two types of voice are motivated by fundamentally different concerns: promotive voice is driven by an opportunity-oriented mindset, while prohibitive voice reflects a risk-avoidance mindset [60,79].

Considering these critical distinctions, it is proposed that the relationship between followers’ ITLMs and voice behavior may differ depending on the type of voice. Specifically, incremental ITLMs, which emphasize beliefs in growth, adaptability, and the possibility to change, may naturally align more closely with promotive voice, given its orientation toward enhancing organizational functioning. At the same time, an alternative perspective suggests that the effect of ITLMs may not differ across voice types. Because both promotive and prohibitive voice are constructive and aimed at improving organizational functioning [60], one could expect that beliefs about leadership malleability would similarly facilitate both forms of voice. If followers view leaders as open to change, they may feel equally encouraged to offer suggestions and to raise concerns.

However, ITLMs may have a stronger effect on promotive voice than on prohibitive voice due to differences in psychological alignment and perceived interpersonal risk. Promotive voice is future-oriented and improvement-focused, making it more consistent with a growth-based construal of leadership as developable and responsive to new ideas. In contrast, prohibitive voice involves pointing out problems or potential failures, which can be perceived as more confrontational and carries greater interpersonal and evaluative risk [80]. Even when followers believe that leadership is malleable, such risk considerations may attenuate their willingness to engage in prohibitive voice. Thus, although incremental ITLMs should promote both forms of voice, their effect is likely to be more strongly expressed in promotive voice, where the behavioral content is more congruent with growth-oriented beliefs and entails lower interpersonal cost.

This reasoning aligns with prior research suggesting that congruence or regulatory fit between implicit beliefs enhances behavioral engagement [35]. For example, individuals with a promotion-focused orientation—which emphasizes gains, advancement, and improvement—are more likely to engage in promotive behaviors due to the regulatory fit between their psychological orientation and the behavioral intent. Likewise, here it is argued that followers with incremental ITLMs will experience a similar regulatory fit with promotive voice, amplifying their willingness to voice suggestions aimed at enhancing organizational functioning. Formally:

*Hypothesis 3:* The type of voice will moderate the relationship between followers’ ITLMs and voice, such that the relationship will be stronger for promotive voice than for prohibitive voice.

## Methods

**Fig 1:**
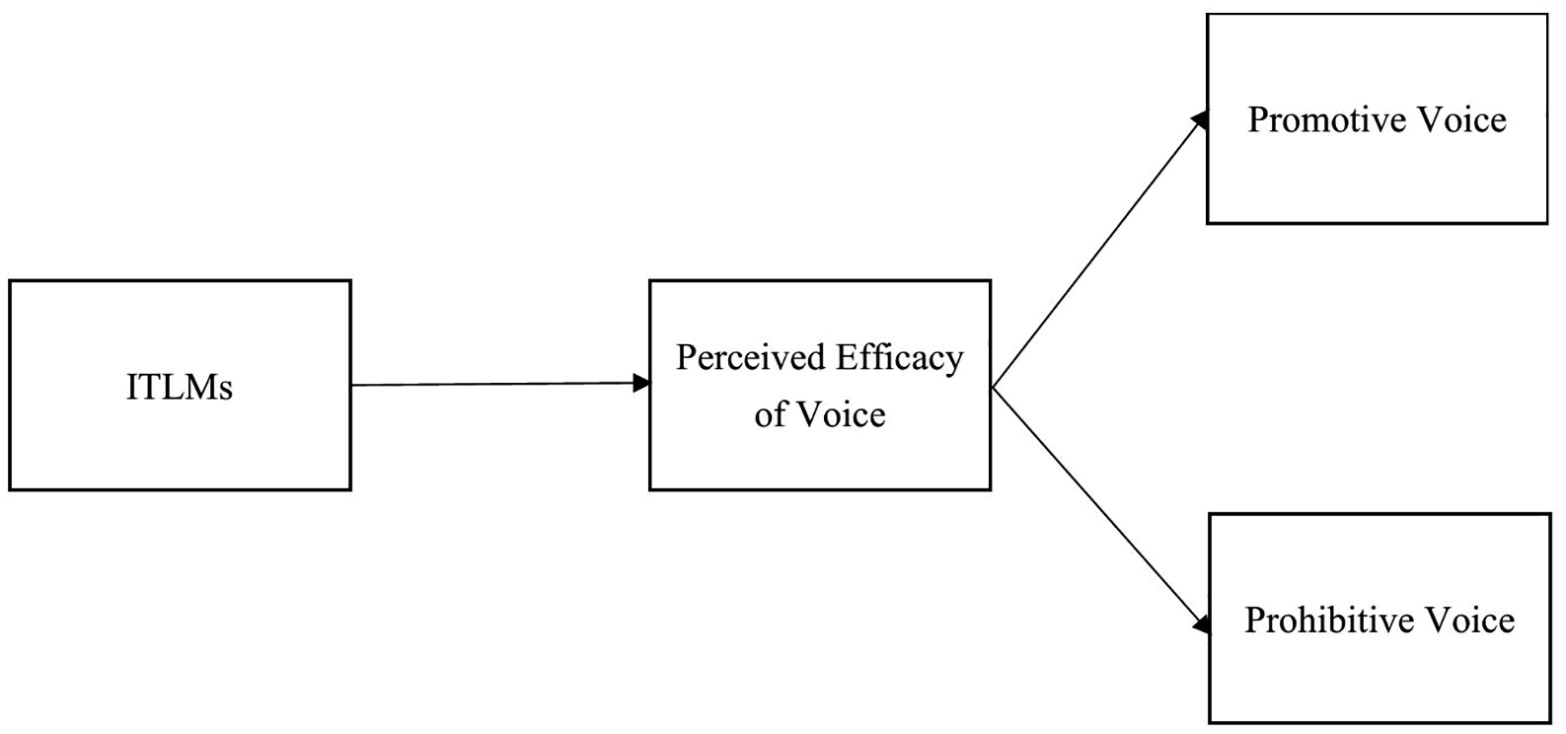
Proposed Model between ITLMs and Voice.

Four studies were conducted to test the hypotheses regarding the relationship between ITLMs and employee voice, with each study designed to build cumulatively on prior findings while addressing specific theoretical and methodological considerations. Fig 1 presents the proposed model. Study 1 employed a scenario-based design to establish the direct relationship between followers’ ITLMs and their general willingness to engage in voice behaviors. Building on this initial evidence, Study 2 moved to a more contextually grounded setting by examining reactions to workplace transgressions and introducing perceived efficacy of voice as a mediator, thus providing deeper insight into the underlying psychological mechanism. Study 3 further refined the theoretical model by incorporating voice type as a moderator, allowing for a test of whether the effects of ITLMs differ between promotive and prohibitive voice. Finally, Study 4 served as a robustness check by replicating the core findings using an alternative scenario-based design while controlling for implicit theories of personality, thus strengthening the construct validity and domain specificity of ITLMs.

### Study 1 Method

The purpose of Study 1 is to establish the utility of considering ITLMs in an organizational context by examining the relationship between ITLMs and employee voice. In this study, participants were asked to complete a measure of ITLMs that we adapted from previous implicit theories measure, and then read a scenario describing a dissatisfactory supervisory relationship. Participants then were asked to indicate the extent to which they would engage in voice. All studies reported in this paper were approved by the University of California, Riverside’s Institutional Review Board (HS 21–162, Amendment #MH31522). Participants indicated informed consent by selecting an agreement button on the online study administration platform (Qualtrics); this alteration of informed consent was approved by the Institutional Review Board. The recruitment period for this study started and ended on May 10^th^, 2022.

### Participants

Two-hundred and fifty participants were recruited from Prolific.com in exchange for $1.00. This was largely a sample of convenience, although the psychological processes underlying the predictions should be broadly applicable to a general population. Nineteen participants were removed from the sample for failing at least one of two attention checks, resulting in a final sample of 231 participants.

### Materials and Procedure

**ITLMs.** To measure ITLMs, a 5-item scale was adapted from the implicit theories of personality scale developed by Chiu and colleagues [17]. Participants indicated their agreement with five statements regarding the fixedness or malleability of leadership characteristics on a 7-point scale ranging from 1 = strongly disagree to 7 = strongly agree. The five items are: “All leaders can change even their most basic leadership characteristics,” “Everybody, no matter who they are, can significantly change their basic leadership characteristics,” “The leadership characteristics of someone are very basic and they can’t be changed very much,” “The way of a leader to lead others is very basic and it can’t be changed very much,” and “Everyone has a certain way to lead others and there is not much that can be done to really change that.” The last three items were reverse scored so that higher ITLM scores indicated a stronger endorsement of an incremental theory (α = .90).

**Employee Voice.** After completing the ITLM measure, participants read the following scenario:

*Imagine that you recently graduated and got a job offer in your dream industry. When you started, the work environment was nice, and the work content was interesting. After several months, however, you’ve encountered some challenges with your supervisor that have made you less enthusiastic about the job. Specifically, you feel as though your supervisor doesn’t give you enough credit for the ideas you contribute to the group, and they frequently pressure you to put in extra hours.*

After reading the scenario, participants were asked to report how they would respond to the dissatisfaction. Specifically, the extent to which individuals would engage in voice was measured using the voice dimension of the Exit, Voice, Loyalty and Neglect framework extensively used in previous research (e.g., [56,80–82]).(Although the focus is on employee voice, the other three potential responses to the dissatisfactory relationship (exit, loyalty, neglect) were also measured. ITLMs were negatively associated with “exit” (r = -.16, p =  .013) and “neglect” (r = -.14, p =  .033). ITLMs were not related to the “loyalty” response (r = -.007, p =  .912).) Four items were used to measure voice (e.g., “I would try to solve the problem by suggesting constructive changes in the office”). Each item was rated on a 7-point scale with 1 = strongly disagree and 7 = strongly agree (α = .76).

### Study 1 Results and Discussion

Means, standard deviations and correlations between all variables are presented in Table 1. A confirmatory factor analysis was conducted using the lavaan package in R. The results indicated that the single-factor model provided an acceptable fit to the data (CFI = 0.985, TLI = 0.963, RMSEA = 0.074, SRMR = .019). All factor loadings were significant and substantial (λs range from.58 to.88). Additionally, we conducted Harman’s single-factor test by entering all measurement items into an exploratory factor analysis without rotation. The first factor accounted for 37% of the total variance, which is below the commonly used threshold of 50%, suggesting that common method bias is unlikely to be a serious concern.

**Table 1:** Means, standard deviations, and correlations between variables for study 1.

Variables	Mean (SD)	1	2	3	4	5
1. ITLMs	5.05 (1.16)	--				
2. Voice	4.86 (1.08)	.22**	--			
3. Exit	3.99 (1.24)	−.16*	−.04	--		
4. Loyalty	3.51 (1.16)	−.01	.03	−.33**	--	
5. Neglect	3.44 (1.32)	−.14*	−.05	.36**	−.07	--

N = 281

* p < .05, ** p < .01.

In Study 1, initial support was found for the first hypothesis that ITLMs are associated with employee voice. With more malleable ITLMs, individuals endorsed directly speaking to their supervisor to mitigate workplace issues. The next study builds on these results.

Consistent with Hypothesis 1, ITLMs were positively associated with the voice measure indicating that employees with higher (i.e., more incremental) ITLMs would engage in more voice behaviors (*r* = .22, *p* = .001). In other words, the greater the extent to which individuals believed that leaders could change their leadership characteristics, the more willing they were to engage in voice. This effect size is small to medium, and is consistent with the size of the correlations that are generally found between individual dispositions (e.g., the Big 5) and voice [83]. Therefore, such effects are meaningful in organizational settings, especially when the effects are aggregated across teams or over time, indicating that fostering malleable ITLMs may yield significant improvements in employee voice, helping organizational communication and decision-making processes.

### Study 2 Method

The results of Study 1 indicate how ITLMs are associated with employees’ behaviors in the context of dissatisfactory supervisory relationships. The purpose of Study 2 is to replicate this finding in the context of actual workplace challenges, and also to explore the mediating role of the perceived efficacy of voice. Participants indicated informed consent by selecting an agreement button on the online study administration platform (Qualtrics); this alteration of informed consent was approved by the Institutional Review Board. The recruitment period for this study started on March 21^st^, 2024 and ended on April 4^th^, 2024.

### Participants

Five hundred full-time employees were recruited from Prolific.com. Although this was a sample of convenience, the sample of working adults is well-suited to testing the predictions about behavior in the workplace. Data were collected via online surveys in two waves separated by a one-week interval. At Time 1, ITLMs, perceived efficacy of voice, and several control variables were collected. At Time 2, employees were asked to recall a challenge they faced with their direct supervisor within the past week and to report their engagement in voice concerning the challenge. Participants were paid $2 for their participation in each wave, for $4 in total if they completed both waves. A total of 369 participants completed both waves (response rate = .73). Of these participants, five failed an attention check question and 22 did not recall any challenge with their direct supervisor; these participants were removed from further analysis.

### Materials and procedure

#### Time 1.

**ITLMs.** ITLMs were measured with the identical 5-item scale used in the previous study. Each item was rated on a 7-point scale with 1 = strongly disagree and 7 = strongly agree (α = .87).

**Perceived Efficacy of Voice.** Perceived efficacy of voice was captured by a 6-item scale developed by Wei and colleagues [37]. Specifically, participants were asked to imagine that they had the opportunity to make some suggestions to their supervisor and were asked how likely it would be for their supervisor to endorse their suggestions if they expressed them in a number of ways. An example way of presenting the suggestion is “Speak up and encourage others in this group to get involved in issues that affect the group.” Each item was rated on a 7-point scale with 1 = very unlikely and 7 = very likely (α = .94).

**Control Variables.** Several variables were controlled for in the analyses. First, the models controlled for both employees’ and supervisors’ genders. Age and tenure of participants were also included as controls because previous studies suggested that they are important factors to predict voice (e.g., [59,84]).

#### Time 2.

**Voice.** One week after completing the initial survey, participants were asked to recall a time during the past week in which they disagreed with their supervisor’s decisions or behavior. After writing a short paragraph describing the experience, participants were asked to rate the severity and frequency of the incident (i.e., “How severe would you evaluate the incident you just recalled?” and “How frequently does this type of incident happen in your experience in your current job?”). Finally, participants reported the extent to which they used voice to address the incident using a scale developed by LePine and Van Dyne [59]. Specifically, participants reported their agreement with a series of statements describing ways in which they could have expressed voice (e.g., “I communicated my opinions about work issues to others in this group even if my opinion was different and others in the group disagreed with me.”). Responses were made on a 5-point scale anchored by 1 = Strongly disagree and 5 = Strongly agree (α = .87).

### Study 2 Results and Discussion

Means, standard deviations, and correlations between all variables are reported in Table 2. We conducted Harman’s single-factor test by entering all measurement items into an exploratory factor analysis without rotation. The first factor accounted for 32% of the total variance, which is below the commonly used threshold of 50%, suggesting that common method bias is unlikely to be a serious concern. Hypothesis 1 predicted that ITLMs would positively relate to employee voice. To test this hypothesis, voice was regressed on the control variables (including incident severity and frequency), and ITLMs. The results of this analysis revealed a significant positive relationship between ITLMs and voice, *b* = .095, SE = .044, *t*(341) = 2.16, *p* = .032.

**Table 2:** Means, standard deviations, and correlations between variables for study 2.

Variables	Mean (SD)	1	2	3	4	5	6	7	8	9
1. ITLMs	5.22 (1.10)	--								
2. Perceived efficacy	5.28 (1.21)	.18**	--							
3. Voice	3.75 (0.92)	.11*	.36**	--						
4. Severity	2.69 (0.79)	.02	.03	−.18**	--					
5. Frequency	1.81 (0.71)	−.05	−.17**	.04	.03	--				
6. Tenure	0.92 (0.27)	−.02	.13*	.17**	−.11*	−.02	--			
7. Age (years)	38.97 (10.66)	−.03	−.06	.04	−.13*	−.12*	.15**	--		
8. Gender	0.51 (0.50)	.06	.08	.15**	−.01	−10	.04	−.10	--	
9. Supervisor Gender	0.58 (0.49)	−.02	.06	.13*	−.02	−.10	.02	.01	.37**	--

N = 342

* p < .05, ** p < .01.

Tenure: Equal to or greater than one year = 1, Less than 1 year = 0; Gender and Supervisor Gender: Male = 1, Other responses (including Female) =0.

Next, Hypothesis 2 was tested, which predicted that the relationship between ITLMs and voice would be mediated by the perceived efficacy of voice. Perceived efficacy of voice was first regressed on the control variables (excluding incident severity and frequency, given that these were measured after we collected the efficacy measure) and ITLMs. The results of this analysis revealed a significant positive relationship between ITLMs and perceived efficacy of voice, *b* = .199, SE = .059, *t*(341) = 3.41, *p* = .001. The mediating role of perceived efficacy of voice was next tested using the PROCESS 5.0 macro in SPSS with 10,000 bootstrapped samples. A significant indirect effect of ITLMs on voice was observed (Indirect Effect = .052, SE = .021, 95% CI = [.015 −.096]), while the direct effect of ITLMs was not significant (Direct Effect = .043, SE = .042, 95% CI = [−.039 −.125]). These results were consistent with Hypothesis 2 and suggest that the positive association between ITLMs and employee voice is mediated by the perceived efficacy of voice.

The results of Study 2 provide additional support for the hypotheses. Consistent with Study 1, a significant positive relationship between ITLMs and engagement in voice was observed. Extending this prior finding, evidence was also found that the positive relationship between ITLMs and employee voice is mediated by individuals’ perceived efficacy of voice.

While the combined results of Studies 1 and 2 provide consistent support for the positive relationship between ITLMs and voice, they are limited in the sense that the global consideration of voice thus far does not address nuances in the way in which employees may address challenges in the workplace. The next two studies explore how the type of voice moderates the relationship.

### Study 3 Method

Study 3 was conducted as an initial test of the prediction that the specific type of voice would moderate the relationship between ITLMs and the willingness to speak up in an organization. Participants indicated informed consent by selecting an agreement button on the online study administration platform (Qualtrics); this alteration of informed consent was approved by the Institutional Review Board. The recruitment period for this study started and ended on March 3^rd^, 2023.

### Participants

Three-hundred and ninety-nine individuals who were currently working full time were recruited from Prolific.com. Although this was a sample of convenience, the sample of working adults is well-suited to testing the predictions about behavior in the workplace. Individuals were paid $2 for their participation. Six individuals failed an attention check and were removed from further analysis resulting in a final sample of 393 participants.

### Materials and procedure

Participants were asked to self-report a series of measurements within the context of their current work environment. Specifically, participants reported their ITLMs, and their tendency to engage in promotive and prohibitive voice in their workplace.

**ITLMs.** ITLMs were measured with the identical 5-item scale used in the previous two studies. Each item was rated on a 7-point scale with 1 = strongly disagree and 7 = strongly agree (α = .92).

**Promotive and Prohibitive Voice.** Participants completed the Promotive voice and Prohibitive voice scales developed by Liang et al. [60] to indicate the extent to which they typically engaged in each type of voice in their workplace. Each type of voice was measured using a 5-item scale with 1 = never and 5 = almost always (10 items in total). An example item for Promotive voice is “I proactively develop and make suggestions for issues that may influence the unit,” while an example item for Prohibitive voice is “I advise other colleagues against undesirable behaviors that would hamper job performance.” The Cronbach’s alphas were 0.91 and 0.86 for Promotive voice and Prohibitive voice, respectively.

**Control Variables.** Controls included both employees’ gender as well as the gender of their direct supervisor, as well as participants’ age and job tenure.

### Study 3 Results and Discussion

Means, standard deviations, and correlations between all variables are reported in Table 3. We conducted Harman’s single-factor test by entering all measurement items into an exploratory factor analysis without rotation. The first factor accounted for 40.7% of the total variance, which is below the commonly used threshold of 50%, suggesting that common method bias is unlikely to be a serious concern. These zero-order correlations suggest that ITLMs are positively related to both Promotive voice (*r* = .21) and Prohibitive voice (*r* = .11), although the relationship is stronger for Promotive voice. The effect size for the relationship with Promotive voice is small to medium, and is consistent with the size of the correlations that are generally found between individual dispositions (e.g., the Big 5) and voice [83]. The effect size for the relationship with Prohibitive voice is considered small, and is smaller than the typical effect size typically found between individual dispositions and voice. As voice behaviors are shaped by multiple constraints and facilitators, looking at the two effect sizes collectively can be meaningful to organizations. In particular, the comparatively stronger association with promotive voice suggests that ITLMs may be more consequential for future-oriented, improvement-oriented behaviors, suggesting that organizations can elicit promotive voice by fostering malleable ITLMs among employees. To test whether the difference in correlations was significant, a test was conducted comparing the difference between two dependent correlations [85,86]. The results of this analysis revealed that the relationship between ITLMs and voice was significantly stronger for Promotive voice compared to Prohibitive voice (*Z* = 2.43, *p* = .015).

**Table 3:** Means, standard deviations, and correlations between variables for study 3.

Variables	Mean (SD)	1	2	3	4	5	6	7
1. ITLMs	5.10 (1.24)	--						
2. Promotive voice	3.69 (1.02)	.21**	--					
3. Prohibitive voice	3.29 (0.99)	.11*	.67**	--				
4. Tenure (years)	5.98 (5.18)	.08	.19**	.10	--			
5. Age (years)	38.25 (11.00)	.02	.08	.01	.45**	--		
6. Gender	0.49 (0.50)	.10*	.09	.13**	.01	−.04	--	
7. Supervisor Gender	0.54 (0.50)	.06	.07	.07	.08	−.04	.38**	--

N = 393

* p < .05, ** p < .01.

Gender and Supervisor Gender: Male = 1, Other responses (including Female) = 0.

**Fig 2:**
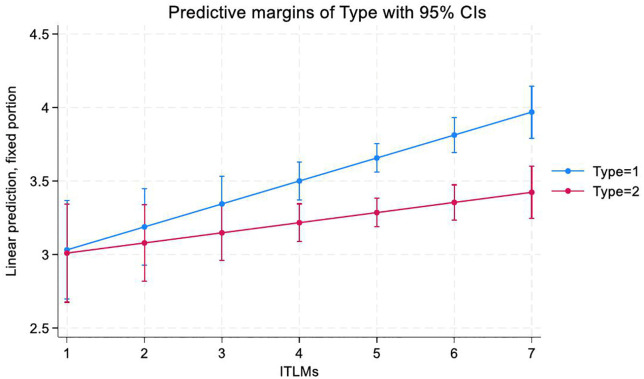
Interaction between ITLMs and Voice Type on Employee Voice. (Type 1 = Promotive, Type 2 = Prohibitive).

To further test our hypotheses, Promotive voice was regressed on the control variables and ITLMs. The results of this analysis revealed a significant positive relationship between ITLMs and Promotive voice, *b* = .160, SE = .041, *t*(387) = 3.88, *p* < .001. Next, Prohibitive voice was regressed on the control variables and ITLMs, revealing a marginally-significant positive relationship between the two variables, *b* = .073, SE = .040, *t*(387) = 1.83, *p* = .068. In order to directly test the prediction that the relationship between ITLMs and voice would be stronger for Promotive voice than for Prohibitive voice (Hypothesis 3), hierarchical linear modelling [87] was employed using the mixed function in STATA 18 to test for a difference between these relationships. The first level, or Level 1, captures the type of voice being considered as a within-subject difference. The second level, or Level 2, captures variables that are constant and do not change when considering voice (i.e., ITLMs, control variables). Hypothesis 3 is tested via the ITLM X Type of Voice between-level interaction. Model 1 in Table 4 presents the base model with the control variables, Model 2 adds ITLMs and Type of Voice as predictors, and Model 3 adds the interaction term. In support of the hypothesis, the coefficient for the interaction term was significant (γ = −.088, *p* = .009), confirming a difference in how ITLMs relate to the different types of voice. The interaction is presented in Fig 2.

**Table 4:** HLM results predicting voice for study 3.

Variable	Coefficient		
	**Model 1**	**Model 2**	**Model 3**
Controls			
Gender	.20*	.18	.18
Supervisor Gender	.04	.04	.04
Tenure	.03**	.03**	.03**
Age	.00	.00	.00
Main Effects			
ITLMs		.12**	.16**
Voice Type		−.38**	.06
Interaction Effects			
ITLMs X Voice Type			−.09**
Wald χ^2^	16.49**	110.22**	118.38**

*Notes: N* = 393; * *p* < .05. ** *p* < .01, Two-tailed; Gender and Supervisor Gender: Male = 1, Other responses (including Female) = 0; Voice Type: 1 = Promotive, 2 = Prohibitive.

The results of Study 3 provide further support for the hypotheses. Consistent with the previous studies, a significant positive relationship between ITLMs and voice was observed. However, extending these prior findings, the relationship between ITLMs and Promotive voice is stronger than the relationship with Prohibitive voice.

### Study 4 Method

The purpose of Study 4 was to replicate and extend the findings of Study 3 by further examining the moderating role of voice type in a new scenario-based context. In addition, in Study 4, the role of general implicit person theories (i.e., can people generally change their basic characteristics) was considered to ensure that the observed effects were specific to ITLMs, providing a more rigorous test of the hypotheses. Participants indicated informed consent by selecting an agreement button on the online study administration platform (Qualtrics); this alteration of informed consent was approved by the Institutional Review Board. The recruitment period for this study started and ended on January 23^rd^, 2025.

### Participants

Three-hundred individuals were recruited from Prolific.com and were paid $1.50 for their participation. This was largely a sample of convenience, although the psychological processes underlying the predictions should be broadly applicable to a general population. Thirteen individuals failed an attention check and were removed from further analysis resulting in a final sample of 287 participants.

### Materials and Procedure

Participants first reported their ITLMs as well as their general Implicit Person Theories (IPTs). Next, they read a scenario describing a challenge they faced with a supervisor. Finally, participants indicated their expected tendency to engage in promotive and prohibitive voice in response to the challenge.

**ITLMs.** ITLMs were measured with the identical 5-item scale used in the previous studies (α = .82).

**IPTs.** General beliefs about the extent to which individuals’ basic characteristics are malleable were measured using a three-item scale developed by Dweck et al. [16]. A sample item is “Everyone is a certain kind of person, and there is not much that they can do to really change that.” Responses ranged from 1 (Strongly disagree) to 7 (Strongly agree). To maintain consistency with the ITLM measure, responses were reverse scored such that higher scores indicated stronger beliefs that basic characteristics are malleable. The scale was adequately reliable (α = .91).

**Promotive and Prohibitive Voice.** After reporting their ITLMs and IPTs, participants read a scenario describing a situation in which their supervisor ignored their input on a project, which subsequently failed. Participants then indicated how they would respond to the situation using the Promotive and Prohibitive voice scales described in Study 3. The Cronbach’s alphas were 0.90 and 0.85 for Promotive voice and Prohibitive voice, respectively.

**Control Variables.** At the conclusion of the study, participants reported their gender and age.

### Study 4 Results and Discussion

**Fig 3:**
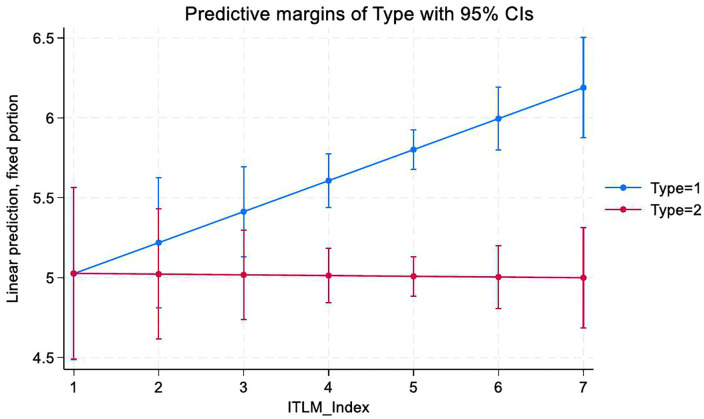
Interaction between ITLMs and Voice Type on Voice. (Type 1 = Promotive, Type 2 = Prohibitive).

Means, standard deviations, and correlations between all variables are reported in Table 5. We conducted Harman’s single-factor test by entering all measurement items into an exploratory factor analysis without rotation. The first factor accounted for 32% of the total variance, which is below the commonly used threshold of 50%, suggesting that common method bias is unlikely to be a serious concern. ITLMs were positively related to Promotive voice (*r* = .17) and were not significantly related to Prohibitive voice (*r* = −.07). The effect size for the relationship with Promotive voice is small to medium, and is consistent with the size of the correlations that are generally found between individual dispositions (e.g., the Big 5) and voice [83]. To test the hypotheses, Promotive voice was regressed on the control variables, IPTs and ITLMs. The results of this analysis revealed a significant positive relationship between ITLMs and Promotive voice, *b* = .169, SE = .065, *t*(286) = 2.59, *p* = .01. Next, Prohibitive voice was regressed on the control variables, IPTs and ITLMs; no significant effect of ITLMs emerged, *b* = .021, SE = .082, *t*(286) = 0.25, *p* = .803. In order to directly test the prediction that the relationship between ITLMs and voice would be stronger for Promotive voice than for Prohibitive voice (Hypothesis 3), hierarchical linear modelling was employed to test for a difference between these relationships. Model 1 in Table 6 presents the base model with the control variables, Model 2 adds ITLMs and Type of Voice as predictors, and Model 3 adds the interaction term. In support of the hypothesis, the coefficient for the interaction term was significant (γ = −.199, *p* < .001), confirming a difference in how ITLMs relate to the different types of voice. The interaction is presented in Fig 3.

**Table 5:** Means, standard deviations, and correlations between variables for study 4.

Variables	Mean (SD)	1	2	3	4	5	6
1. ITLMs	4.86 (1.22)	--					
2. Promotive voice	5.77 (0.95)	.17**	--				
3. Prohibitive voice	5.01 (1.21)	−.07	.56**	--			
4. IPTs	4.07 (1.60)	.72**	.11	−.07	--		
5. Age (years)	40.97 (12.86)	.12*	−.11	−.25	.04	--	
6. Gender	0.47 (0.50)	−.03	.02	.09	0.02	−.05	--

N = 287

* p < .05, ** p < .01.

Gender: Male = 1, Other responses (including Female) = 0.

**Table 6:** HLM results predicting voice for study 4.

Variable	Coefficient		
	Model 1	Model 2	Model 3
Controls			
IPTs	.01	−.04	−.04
Gender	.11	.12	.12
Age	−.02**	−.02**	−.02**
Main Effects			
ITLMs		.09	.19**
Voice Type		−.77**	.20
Interaction Effects			
ITLMs X Voice Type			−.20**
Wald χ^2^	14.52**	172.54**	198.07**

*Notes: N* = 287; * *p* < .05. ** *p* < .01, Two-tailed; Gender: Male = 1, Other responses (including Female) = 0; Voice Type: 1 = Promotive, 2 = Prohibitive.

## General discussion

This paper began by asking whether leadership is a bundle of relatively fixed traits, or is a skill that can be developed over time. A long history of leadership research supports both perspectives, indicating validity in a range of beliefs regarding the fixedness or malleability of leadership characteristics. The current research focused on these beliefs – referred to as Implicit Theories of Leadership Malleability (ITLMs) – and examined how they shape important workplace behaviors, particularly employee voice. Building on the broader framework of implicit theories [9], this research distinguished between individuals who view leadership characteristics as stable and unchangeable (entity theorists) and those who view them as dynamic and improvable (incremental theorists).

These findings contribute to a growing reorientation in leadership research by shifting the role of followers from passive recipients of leadership to active participants in shaping leadership itself. Specifically, when followers hold incremental ITLMs, they are more likely to view leadership characteristics as responsive to input, feedback, and interaction. This belief fundamentally alters the nature of the leader–follower relationship: voice behavior becomes not merely a reaction to leadership, but a mechanism through which followers attempt to influence and potentially develop their leaders. In this sense, leadership is not only enacted by leaders but is co-constructed through followers’ proactive engagement. This perspective extends existing followership theories by highlighting that followers’ implicit beliefs about leadership malleability serve as a critical cognitive foundation that enables or constrains their willingness to participate in this co-construction process.

The current work makes a number of important theoretical contributions. First, this research advances the literature on followership by identifying a previously overlooked cognitive mechanism that shapes how followers engage with leadership. While prior work has emphasized followers’ traits, identities, and role orientations [34,53], it has paid less attention to how followers construe leadership itself. The current findings suggest that followers’ beliefs about the malleability of leadership characteristics fundamentally shape their behavioral responses, particularly their willingness to engage in voice. Importantly, this shifts the focus from “what followers are” to “how followers think about leadership,” thereby introducing ITLMs as a key cognitive lens through which leader–follower dynamics unfold.

Second, these findings extend leadership theory by challenging the traditional unidirectional view in which leaders influence followers. Much of the leadership literature implicitly assumes a top-down process, where leader traits and behaviors shape follower outcomes. In contrast, the current results suggest that this influence is contingent on followers’ beliefs about whether leadership itself is malleable. When followers perceive leadership as developable, they are more willing to engage in behaviors—such as voice—that have the potential to influence and reshape leadership over time. This implies a more dynamic and reciprocal process, in which leadership is continuously constructed through interaction rather than imposed unilaterally. By introducing ITLMs into this process, the current work provides a cognitive mechanism that explains when followers are likely to participate in shaping leadership, thereby enriching relational and socially constructed views of leadership.

Finally, these findings contribute to the voice literature by offering a more nuanced understanding of how different forms of voice are linked to underlying cognitive beliefs. Although both promotive and prohibitive voice are constructive, these results suggest that ITLMs more strongly facilitate promotive voice than prohibitive voice. This asymmetry provides insight into the nature of follower-driven influence in organizations. Specifically, incremental ITLMs appear to encourage a form of engagement oriented toward developmental co-construction, where followers contribute ideas to improve and evolve leadership and organizational practices, rather than corrective or confrontational forms of input. In this sense, these findings suggest that beliefs about leadership malleability do not simply increase voice behavior, but shape the manner in which followers seek to influence leadership, favoring growth-oriented contributions over problem-focused corrections.

In addition to its theoretical implications, the current research has important managerial implications. Employees’ voice behavior is critical to organizational success and to leader-follower relationships [88,89]. However, in the workplace, people are often reluctant to express their real opinions because of various concerns. For example, previous research focusing on personal dispositions had identified various antecedents which make people hesitate to engage in voice behaviors, such as low core self-evaluations [90], low status [91,92], as well as gender differences [93]. Additionally, leaders’ behaviors and personalities are also important to employees’ willingness to speak up. For instance, Guzman & Fu [94] found that when leaders and followers both held low power distance values, there was greater voice behavior. Contextual factors have also been considered. Ng et al. [95] suggested that the relationships among coworkers are considered when employees decide whether they would speak up, specifically, employees who believe that they are respected by their coworkers are motivated to voice more. Building on this body of work, these findings suggest that managers should pay attention not only to structural and interpersonal conditions that facilitate voice, but also to employees’ underlying beliefs about leadership itself. Managers should recognize that fostering beliefs in the malleability of leadership may not only increase employees’ willingness to speak up, but also encourage a more developmental form of engagement in which employees actively contribute to improving leadership and organizational functioning over time.

### Limitations and future directions

Although the current research provides a robust set of results suggesting the association between ITLMs and employee voice, there are limitations that should be acknowledged, which in turn point to important directions for future work.

First, these findings are constrained by the nature of our research designs. Although multiple studies were conducted with complementary approaches, the results relied on cross-sectional data, which limits the ability to draw strong causal inferences. While the scenario-based designs help establish internal validity, they may not fully capture the dynamic and evolving nature of leader–follower interactions in real organizational settings. Future research would benefit from longitudinal designs that track how followers’ ITLMs and voice behaviors co-evolve over time, as well as field experiments or intervention studies that manipulate beliefs about leadership malleability to establish causal effects more directly.

Second, all variables in the current research were self-reported, raising the possibility of common method bias. To mitigate this concern, we incorporated several remedies at the design and analysis stage. Specifically, we temporally separated the measurement of key variables across different waves, used well-validated scales with distinct item wording to minimize ambiguity and item overlap, and provided Harman’s single-factor test for every study. Although the use of different study designs and post-hoc analysis mitigates this concern to some extent, reliance on a single source may still inflate observed relationships. Future research should adopt multi-source designs, such as combining follower-reported ITLMs with supervisor-rated voice behavior or peer evaluations, to capture enacted behavior rather than intentions. Peer evaluations may serve as an additional source to assess informal voice or day-to-day speaking up that may not be fully visible to supervisors. Taken together, incorporating behavioral or archival indicators of voice would further strengthen measurement validity. In addition, future studies can incorporate experimental design to avoid common method bias through manipulating followers’ ITLMs.

Third, the measurement approach, while grounded in prior implicit theories research, may not fully capture the complexity of leadership-related beliefs in organizational contexts. ITLMs reflect generalized beliefs about the malleability of leadership characteristics, but followers’ interpretations of leadership may also be shaped by more proximal relational constructs. For example, leader–member exchange (LMX) and other relationship-based variables may influence both how followers perceive leadership and their willingness to speak up. Although the findings remained robust with basic demographic controls, future research should incorporate a broader set of theoretically relevant controls, such as LMX, leadership style, or psychological safety, to better isolate the unique contribution of ITLMs.

Fourth, our model may omit some relevant individual difference variables that could account for additional variance in voice behavior. While ITLMs capture beliefs about the malleability of leadership characteristics, individuals’ broader self-related beliefs, such as self-confidence, reflecting a general sense of one’s capability, and perceived control, reflecting beliefs about one’s ability to influence outcomes, may also shape their willingness to speak up. These constructs may interact with ITLMs by influencing whether individuals feel empowered to translate their beliefs into action. Although our findings remained robust with standard controls, future research should incorporate these more general belief constructs to better disentangle the unique explanatory role of ITLMs and to provide a more comprehensive understanding of the cognitive antecedents of voice behavior.

Fifth, the generalizability of our findings may be bounded by the cultural and contextual settings in which the data were collected. Beliefs about authority, hierarchy, and the malleability of personal attributes vary across cultural contexts, which may shape both ITLMs and voice behavior. For instance, in high power distance contexts, even followers who endorse incremental ITLMs may be less likely to engage in voice due to stronger norms around deference to authority. Future research should conduct cross-cultural comparisons to examine how cultural values moderate the relationship between ITLMs and voice, thereby clarifying the boundary conditions of our theoretical model.

Finally, the current research opens several avenues for future inquiry into the dynamic nature of ITLMs. Although implicit theories are conceptualized as relatively stable yet malleable, little is known about how beliefs about leadership develop over time. Future studies could examine whether ITLMs can be shaped through targeted interventions, such as leadership training programs, feedback systems, or exposure to developmental leadership models. Longitudinal and experimental designs would be particularly valuable in examining how changes in ITLMs influence not only voice behavior but also broader patterns of leader–follower interaction and organizational adaptation. In addition, future research could explore the joint effects of leaders’ and followers’ ITLMs, as well as their alignment or misalignment, to provide a more complete understanding of how shared or divergent beliefs shape relational dynamics in organizations.

## Conclusion

This paper applies the implicit theory framework to the leadership domain, and explores how followers’ implicit theories of leadership malleability associate with their willingness to engage in voice. Consistent links are found between incremental beliefs regarding leadership malleability and voice, particularly for promotive voice directed toward growth and improvement.
